# Experimental Optimization of Process Parameters in CuNi18Zn20 Micromachining

**DOI:** 10.3390/mi12111293

**Published:** 2021-10-21

**Authors:** Andrea Abeni, Alessandro Metelli, Cristian Cappellini, Aldo Attanasio

**Affiliations:** 1 Department of Mechanical and Industrial Engineering, University of Brescia, Via Branze 38, 25123 Brescia, Italy; andrea.abeni@unibs.it (A.A.); alessandro.metelli@unibs.it (A.M.); 2 Faculty of Science and Technology, Free University of Bolzano, Piazza Università 1, 39100 Bolzano, Italy; cristian.cappellini@unibz.it

**Keywords:** micromilling, process optimization, ANOVA, surface finishing

## Abstract

Ultraprecision micromachining is a technology suitable to fabricate miniaturized and complicated 3-dimensional microstructures and micromechanisms. High geometrical precision and elevated surface finishing are both key requirements in several manufacturing sectors. Electronics, biomedicals, optics and watchmaking industries are some of the fields where micromachining finds applications. In the last years, the integration between product functions, the miniaturization of the features and the increasing of geometrical complexity are trends which are shared by all the cited industrial sectors. These tendencies implicate higher requirements and stricter geometrical and dimensional tolerances in machining. From this perspective, the optimization of the micromachining process parameters assumes a crucial role in order to increase the efficiency and effectiveness of the process. An interesting example is offered by the high-end horology field. The optimization of micro machining is indispensable to achieve excellent surface finishing combined with high precision. The cost-saving objective can be pursued by limiting manual post-finishing and by complying the very strict quality standards directly in micromachining. A micro-machining optimization technique is presented in this a paper. The procedure was applied to manufacturing of main-plates and bridges of a wristwatch movement. Cutting speed, feed rate and depth of cut were varied in an experimental factorial plan in order to investigate their correlation with some fundamental properties of the machined features. The dimensions, the geometry and the surface finishing of holes, pins and pockets were evaluated as results of the micromachining optimization. The identified correlations allow to manufacture a wristwatch movement in conformity with the required technical characteristics and by considering the cost and time constraints.

## 1. Introduction

The horology industry represents one of the major manufacturing sectors for mi-cromachining. In particular, micromilling and microdrilling finds several applications in the manufacturing of the mechanical watches. Although quartz watches based dominate the watch market, the business turnover related to mechanical watches is meaningfully higher [1]. The standard size of a wristwatch movement is very limited, with a diameter ranging from 17 mm to 36 mm and a thickness lower than 4 mm. In the low volume of the caliber are assembled some hundreds of miniaturized components, such as wheels, shafts, levers, screws, springs and bushing. The components are mounted inside the case of the mechanical movement, which is composed by a main plate and the bridges. All the parts have pockets, micro-holes and micro-pins to mechanically constrain the miniaturized components. The micromachining of these features must comply with tight dimensional and geometrical tolerances. Furthermore, the surface finishing assumes a crucial role in order to reduce the frictional effects and to guarantee adequate visual appearance.

Among the manufacturing processes, micromilling is one of the most flexible and fastest way to produce complex tridimensional microfeatures with high dimensional accuracy [2]. It can be successfully applied to a wide variety of materials by using micro-scaled mill with diameters in a sub-millimeter range with different coatings [3]. Micromilling is a subtractive process characterized by a contact between the tool cutting edge and the workpiece along a defined path. As in conventional-size milling, the cutting speed, the feed rate, and the depth of cut are the most meaningful process parameters. The research of the best combinations of these parameters is crucial to guarantee high precision, flexibility, excellent surface finishing, tight geometrical and dimensional tolerances with a low material scrap in the micrometric magnitude scale [4,5]. The selection of the process parameters must also consider the tool deflection effect, defined also as tool run-out effect [6]. The high ratio between the tool length and its diameter results in a drastic reduction of the tool shank section modulus. Form and feature geometric errors on the machined component and distortion of cutting forces are the most undesired effects of tool deflection [7,8]. The component assembly and the product functioning are strongly correlated to the machining optimization. Surface roughness, holes and pins geometrical accuracy (dimension and circularity), burrs extension and their distribution must be controlled by optimizing the process parameters [9].

In contrast with conventional scale milling, when performing a micromilling operation, particular attention must be paid to size and surface effects, since they strongly affect the chip formation and the related surface quality. In micromilling, in fact, the dimensions of uncut chip thickness and work material grain size are comparable. Grain-size effects influence the chip typology, leading to a quasi-shear extrusion chip and cutting force increase, when the grain size decreases [10,11]. Moreover, this process is characterized by a relatively small ratio between the depth of cut (*DOC*) and the cutting-edge radius (*r*_0_) [12]. It results in negative rake angles, increasing the compressive stresses on the work material that reduces the chip brittle fracture, by enhancing the material plasticity. An increment of the negative rake angle favorites the contact between the back cutting surface and the work-piece surface, altering the shearing cutting mode into extrusion cutting, ploughing, or roughing in which the workpiece material undergoes to scraping, squeezing, and grooving instead of been correctly cut [13,14,15]. Additionally, indentation effects can take place, consistently rising the surface hardness and cracks propagation [16,17]. The amount of residual chips at the bottom and side of the micro-slot may affect the surface quality. These effects may result in higher size and number of burrs and rapid wear of the micro-milling tool [18]. The burrs can be defined as the residual material that overhangs outside the workpiece edge after machining. Several deburring processes are currently employed in the industrial processes, but the removal may require several minutes and damage the machined features. Therefore, the burr minimization on microparts during micromilling is more desirable and advisable compared with the deburring [19]. Several investigations were published about the thematic of the burr reduction [20,21,22]. The effect of the process parameters was quantitively or qualitatively computed by considering different work-piece materials, such as Inconel 718 [23], stainless steel AISI 316L [24] and Ti-6Al-4V [25]. The experimental researches revealed different behaviors for different materials.

On the other hand, there is a lack of information on machinability at microscale of the materials commonly employed in watch movements. Nickel silver, brass and gold are some of the materials used to produce main plate and bridges, but the CuNi18Zn20 is probably the most common alloy in the horology field due to its grey appearance, higher mechanical properties, and better corrosion resistance than other brass alloys [26]. CuNi18Zn20 is a free lead brass alloy characterized by a single α-phase microstructure. Several studies were performed on the machinability of lead free brasses, revealing their difficult to be machined due to the high ductility of α-phase, leading to extended burrs formation and low surface quality, when conventional cutting processes are performed [27,28]. Amongst these, the results related to the analysis of CuZn38As brass alloy are of great interest, since it is mainly composed by an α-phase microstructure (98%) and shows a machinability comparable with CuNi18Zn20 [29]. This study underlined that to reduce the machined surface roughness and to achieve better dimensional tolerances, low feed rate must be employed. However, although a wide documentation can be found on their machinability, in literature there are not papers which deals with the issues which afflicts the watch making industry, where micromachining conditions are employed. In this case, low feed rate and depth of cut, combined with high cutting speed are suggested [30,31,32], but no bibliographic references concerning the processing of CuNi18Zn20 can be found. This paper deals with the optimization of finishing operations in micromachining of microfeatures on CuNi18Zn20 samples. The optimization process was performed using a microtool with a standard geometry, therefore the obtained results can aid the manufacturers in the choice of the process parameters; An experimental approach was adopted by designing a prototype which included some ad hoc features in a limited volume. The selected features consist in microholes, micropins and pockets. In watchmaking industry, microholes are necessary to couple the bridges and the main plate with the jewels that binds the shafts of the wheels. The diameters usually are equal to few hundreds of micrometers while the required tolerances are reduced to few tens of micrometers to ensure the correct mechanical coupling. Pins ensure the fixing between the main plate and the bridges, and their dimension is comparable with the diameter of the holes. The cylindricity of pins is an imperative requirement to avoid an uncorrected interference with the holes into the bridges. Finally, pockets are fabricated to house the wheels in the caliber structure and their depth is crucial for a corrected wheels positioning.

The micromachining of the listed features was repeated several times by varying the process parameters on three levels and testing all the possible combinations. The outputs were evaluated in terms of diameter, circularity and cylindricity of pins and holes. Moreover, *S_a_*, *S_sk_*, and *S_ku_* roughness parameters of the pocket surfaces were measured in order to examine the surface quality [33]. The correlation between process parameters and outputs was analyzed by means of Analysis of Variance (ANOVA) technique. The burrs extension was checked by using a multifocal 3D microscope. Finally, an optimal parameters set was selected by considering also the time production necessary to fabricate each prototype.

## 2. Materials and Methods

**Figure 1:**
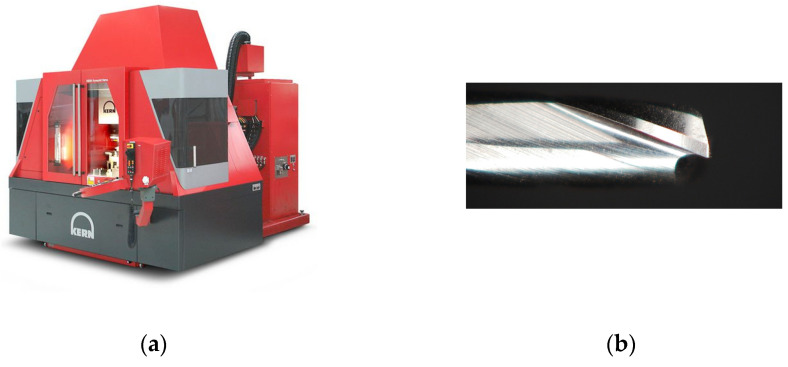
The KERN Pyramid Nano Ultraprecision machining center (**a**); the SECO 905L008-MEGA-T micromill (**b**).

Micromachining tests were executed by using a five axis Nano Precision Machining Centre KERN Pyramid Nano equipped with a Heidenhain iTCN 530 numeric control. It is visible in Figure 1a. The spindle reaches a maximum rotational speed of 50 krpm with a maximum torque of 1.5 Nm. The machining center operates in a controlled environment with a temperature of 20 ± 0.5 °C and humidity of 35% to guarantee the maximum accuracy and precision [34]. The micromill features are summarized in Table 1.

**Figure 2:**
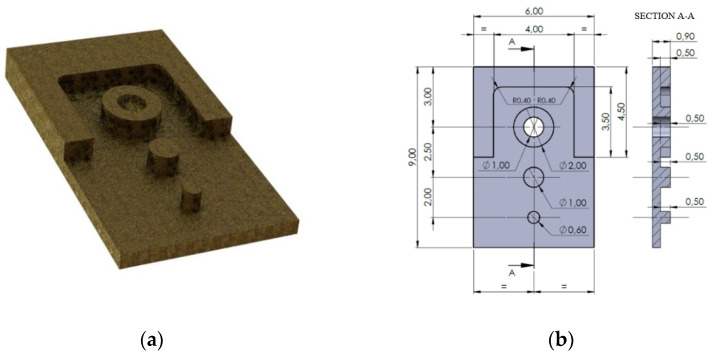
The micromachined prototype: (**a**) a 3D render; (**b**) the 2D dimensioned drawing.

A SECO 905L008-MEGA-T sample is visible in Figure 1b. The actual diameters and the actual cutting-edge radius were measured by using a 3D multifocal microscope Hirox RH-2000 which guarantees an accuracy of 0.8 μm. The micromachining was performed on CuNi18Zn20 samples. The alloy is characterized by brightness (i.e., the intensity of light emanating from the machined surface which depends on the final roughness), good elasticity, excellent mechanical resistance and good machinability and it finds wide applications in watchmaking industry. The experimental tests consist in the micromachining of a self-designed prototypal geometry. It is shown in Figure 2. The prototype dimension is 9 mm × 6 mm × 0.9 mm and it was manufactured in two steps:

- A rough machining to cut a block from a sheet with a thickness of 1.2 mm. A 3 mm diameter end-mill was utilized to machine the blank.
- The two-flutes 0.8 mm diameter micromill was employed to machine a 0.5 mm thickness pocket by leaving three pins. The diameters of the pins are Φ 0.6 mm, Φ 1 mm and Φ 2 mm. The same micromill was employed to machine a through-hole with a diameter equal to Φ 1 mm on the Φ 2 mm pin. The CAM set allowed to perform a normal attack to the surface of the workpiece.

The listed features were identified as critical parts commonly micromachined on a wristwatch caliber. The prototypes were machined 27 times by changing the depth of cut (*DOC*), the feed per tooth (*f_Z_*) and the cutting speed (*V_C_*) on three levels (Table 2) in a 3^3^ complete factorial plan. Table 3 summarizes the values of the process parameters. Machining was performed in lubricated condition by using emulsified oil and monitoring the tool wear to ensure that is neglectable.

Micromachining was performed with the final purpose of identifying the best process parameter set, by considering the features of the machined workpiece and the constraints related to the productivity. Production time oscillates between 74 s and 686 s with an average time of 224 s. The data are collected in Table 3.

Low feed rates combined with low depths of cut determine very low productivity. Moreover, the 80% of the tests have a production time between 74 s and 283 s, which can be considered a reasonable processing time. The process parameters sets 1, 2, 4, 5, 9 can not be considering efficient, however the tests were performed in order to have a benchmark for the quality of the other machined samples.

A coordinate measuring machine, a multifocal 3D microscope and an autofocus 3D laser probe were employed to check the quality of machining on the samples. The CMM optical machine Mitutoyo Quick Scope was employed to measure the diameter and the circularity of the pins and the holes. The instrument guarantees an accuracy of 2.5 μm and each measure was repeated three times by changing the magnification. Hirox RH-2000 multifocal microscope was utilized to perform a visual inspection of the burrs and the shape of the features. The pin conicity was measured with the microscope. Lastly, in order to achieve information related to the machined surface quality, different roughness measurements were performed by means of the autofocus 3D laser probe Mitaka PF-60. In particular, the measured roughness parameters are *S_a_*, *S_sk_*, and *S_ku_* that are the extension to a surface of the linear parameters *R_a_*, *R_sk_*, and *R_ku_* respectively. Roughness *S_a_* is defined as the difference in height of each point compared to the arithmetical mean of the surface, as reported in Equation (1).

(1) $$S_{a}=\frac{1}{A}\iint \left|z\left(x,y\right)\right|dx\;dy$$

Roughness *S_sk_* (skewness), reported in Equation (2), is calculated as the quotient of the mean cube value of height and the cube of the mean square deviation of the height *S_q_* respect to the arithmetical mean of the surface

(2) $$S_{sk}=\frac{1}{S_{q}^{3}A}\iint z^{3}\left(x,y\right)dx\;dy$$

where *S_q_* is (Equation (3))

(3) $$S_{q}=\sqrt{\frac{1}{A}\iint \left|z^{2}\left(x,y\right)\right|dx\;dy}$$

Roughness *S_ku_* (kurtosis), reported in Equation (4), is defined as the ratio between the mean fourth power of height and the fourth power of *S_q_* within the sampling area.

(4) $$S_{ku}=\frac{1}{S_{q}^{4}A}\iint z^{4}\left(x,y\right)dx\;dy$$

Even if the measurements of these roughness parameters are time consuming, they represent more detailed data about the whole surface finishing. *S_a_* furnish a global overview of the surface quality since, considering its definition, it is not significantly affected by scratches, contamination, and measurement noise. On the other hand, *S_sk_* and *S_ku_* values permit the assessment of the shape of valleys and peaks on the surface. In particular, *S_sk_* leads to the estimation of the asymmetry of height discrepancies respect to the mean plane, being it suitable for abrasion evaluation in case of sliding surfaces. Negative values of *S_sk_* suggest the surface is mainly constitute of valleys, that are useful for lubrication purposes, while positive *S_sk_* values indicate that primarily peaks and asperities characterize the surface. A more detailed measure of peaks and valleys sharpness is provided by *S_ku_* where, a smooth surface is individuated by a value lower than 3, while above this value the surface shows sharp asperities. The high precision along the surfaces requested to guarantee accurate contact between the component faces in horology, makes the evaluation of roughness parameters an attractive technique for quality assessment in watchmaking industry [35]. The sampling area *A* employed in these measurements was selected on the base of the pocket and it was equal to 1 mm × 1 mm.

## 3. Results

### 3.1. Qualitative Analysis

**Figure 3:**
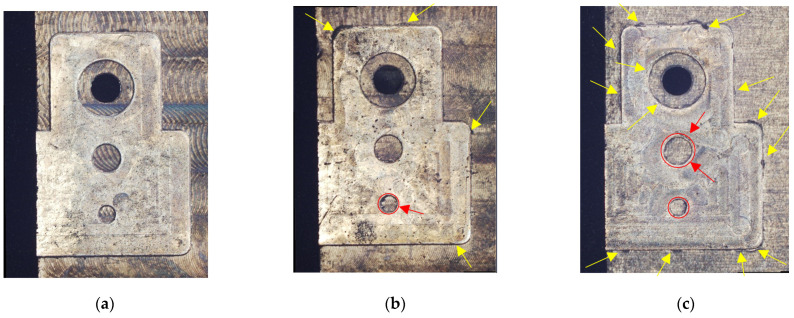
Quality of the machined surface for three different tests: (**a**) Test 1; (**b**) Test 9; (**c**) Test 27.

An initial qualitative inspection of the surface of the machined samples was accomplished employing the microscope Hirox RH-2000 with a magnification of X250. In Figure 3 the effects of the process parameters variation on the quality of three significant examples is shown (Test 1, 9 and 27).

The sample in Figure 3a, namely Test 1, was obtained employing the minimum value of depth of cut, feed per tooth and cutting speed. The machined surface results to be of excellent quality since there is no burrs formation and the circularity of holes and pins is optimal. The values of these latter, in fact, have a deviation from the nominal value that is lower than 2 μm.

In Figure 3b, the surface aspect of Test 9 is observable. This sample was milled utilizing the same depth of cut and feed per tooth, but the cutting speed was raised from 30 m/min to 50 m/min. As a consequence, more burrs are present respect to Test 1 (marked by the yellow colored arrows), but in any case, their reduced dimensions do not prejudice the mechanical coupling between parts.

The last sample in Figure 3c is related to Test 27 in which the maximum values of depth of cut, feed per tooth and cutting speed were applied. In this case, a wide diffusion of burrs with dimensions that are not negligible is present. Similar distributions of the burrs were detected in the samples of Test 25, and Test 26. The totality of these three tests were performed by employing a value of the depth of cut of 150 µm and a value of the feed per tooth of 15 µm, hence the application of the maximum value of depth of cut in conjunction with the maximum value of the feed per tooth is not suitable to answer to surface quality requirements. Moreover, the dimensions and the shape of pins and holes for Test 27 are not acceptable. In particular, the shape of the machined pin with a diameter of 0.6 mm is irregular, as marked by the red colored circles and arrows. Analogous defects were observed in the samples processed with a feed per tooth of 10 µm in conjunction with a cutting speed of 40 m/min and 50 m/min, and in the samples machined with a feed per tooth of 15 µm irrespective to the cutting speed.

Following this preliminary qualitative examination, a quantitative evaluation of the quality of the machined feature results was conducted. In order to correlate the machining parameters with the experimental measurements, analyses of variance of these latter were performed as well.

### 3.2. Quantitative Analysis, ANOVA and Optimization

Quantitative measurements of diameters of the three pins and of the hole, and their circularities were achieved by means of the CMM machine Mitutoyo Quick Scope, while Hirox RH-2000 microscope was employed for estimating the conicity of the pins. Amongst the realized features, the pins with a nominal diameter of 1 mm and 2 mm presented low variability and high precision, whereas the manufacture of the hole and of the smaller pin exhibited the most important criticalities.

With the aim of evaluating how the employed process parameters affect the specimen quality, an analysis of variance (ANOVA), for each measured machined feature, namely hole diameter, the smaller pin diameter, circularity, and conicity, and roughness parameters, was performed. This technique allows to understand the influence of the variation of the process parameters on material machinability as well, and it is mandatory to optimize the micromilling operation.

**Figure 4:**
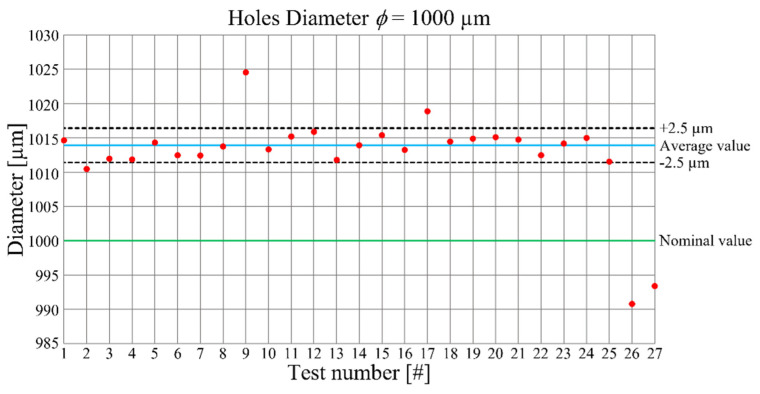
Hole diameters as a function of the performed tests.

The hole diameter values for each test are visible in Figure 4. In this graph, the nominal and the average values of the diameter are also plotted. The average diameter is equal to 1.014 mm and, around it, considering the accuracy of ±2.5 µm of the CMM machine, upper and lower bounds were constructed. For this reason, the points that are contained in the band can be approximated with the mean value. The offset between nominal and average value is equal to 14 µm, indicating a low accuracy of the process with a percentage error of 1.4 %. On the other hand, the data are good placed inside the band, underlining high repeatability but, due to their positioning, the dependency of the hole diameter from the process parameters is not deductible. A deeper knowledge of the dependency was achieved by the related ANOVA, which results are reported in Table 4.

**Figure 5:**
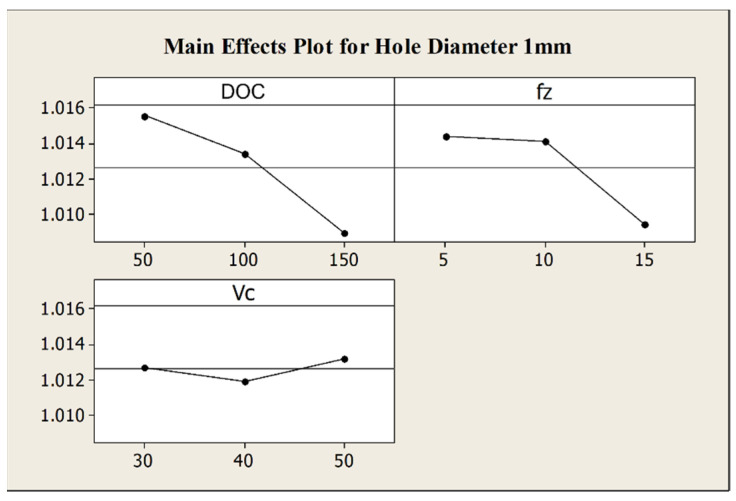
Main effects plot for the hole diameter.

The performed ANOVA underlines that the hole diameter is mainly affect by the depth of cut and by its interaction with the feed per tooth, while the single effect of this latter is of low influence. In particular, a reduction of both depth of cut and feed per tooth leads to an increase of the hole diameter, as it is observable from the relative main effects plot in Figure 5 in which the negligible effect of the cutting speed is visible as well. The reason of this behavior is imputable to the tool run-out effect. An increase in depth of cut and feed per tooth, in fact, causes an increment of the cross-sectional area of the chip with a rise in the cutting forces. Due to the small ratio between tool diameter and tool length, in the presence of high cutting forces, the deflection of the tool axis respect to the spindle axis becomes not negligible [6], carrying out to a reduction of an internal feature as the hole diameter.

**Figure 6:**
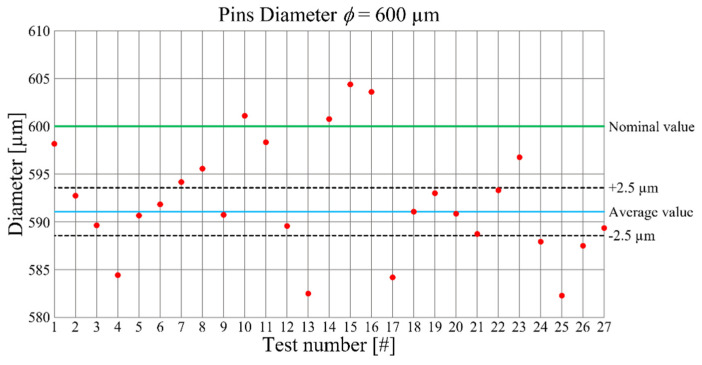
Pin diameters as a function of the performed tests.

Figure 6 shows the pin diameter measurements as a function of experimental test number. The average pin diameter is equal to 0.593 mm with an offset from the nominal value of 7 µm. The value of the percentage error is 1.2% which is comparable with the one related to the hole diameter. The precision of the pins is lower than the precision of the holes.

The analysis of variance of the pin diameter (Table 5) does not show a clear dependency of if from the process parameters. For this reason, the main effects plot is not reported, and the cutting conditions must be further investigated by increasing the range of variability.

**Figure 7:**
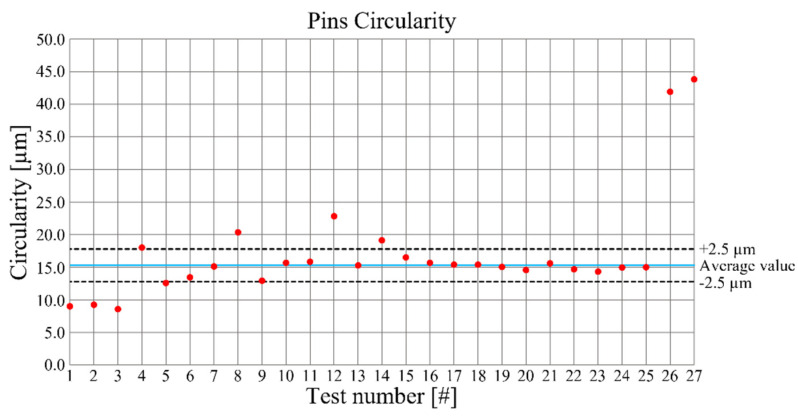
Pin circularities as a function of the performed tests.

Pin circularity is depicted in Figure 7 where a small dispersion of the experimental measurements, except the ones at higher depth of cut, feed per tooth and cutting speed, is visible, indicating an acceptable precision in the analyzed process parameters variation range.

**Figure 8:**
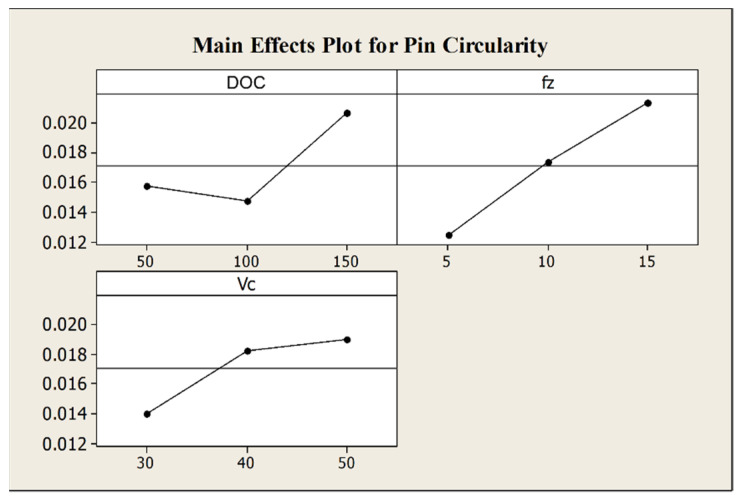
Main effects plot for pin circularity.

As underlined by the ANOVA results in Table 6, the pin circularity is heavily affected by the feed per tooth and its interaction with the depth of cut. In particular, the main effects plot in Figure 8 shows that pin circularity increases when feed per tooth and depth of cut grow.

**Figure 9:**
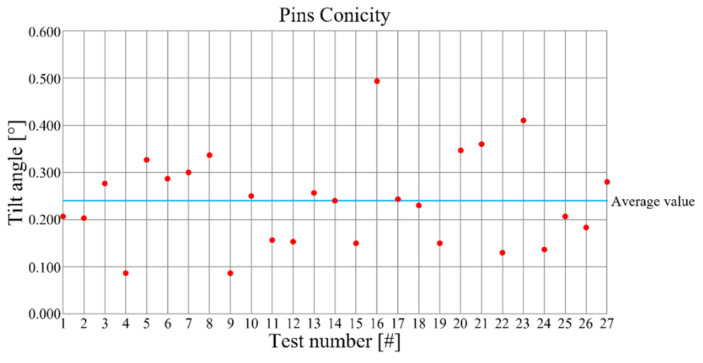
Pin conicities as a function of the performed tests.

The conicity of the pins was assessed by the evaluation of the tilt of the lateral surface in comparison to the normal direction respect to the pocket surface. The related tilt angle ranges between 0° and 0.5° for all the tests, as shown in Figure 9.

The pin conicity does not shows any dependencies from the considered process parameters (ANOVA results in Table 7). Also in this case, the influence of cutting parameters need to be further investigated and the main effects plot is not shown. In any case, the value of the inclination angle of the lateral surface does not prejudice the mechanical coupling of pin with other parts.

The machined surface was evaluated analyzing the roughness parameters *S_a_*, *S_sk_*, and *S_ku_* that provide information about the overall quality and sharpness of the asperities.

All the considered roughness parameters result to be independent from the cutting speed while, varying from the specific parameter, the depth of cut and the feed per tooth have noticeable effects, as highlighted from the analysis results in Table 8, Table 9 and Table 10.

**Figure 10:**
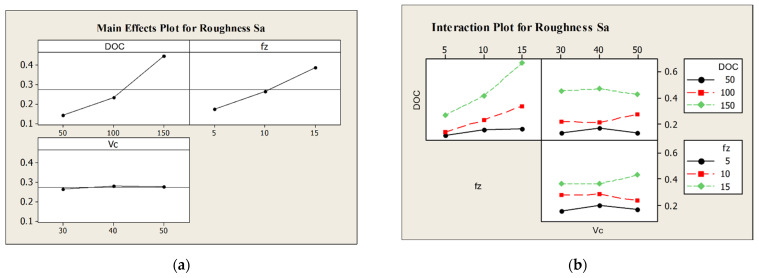
(**a**) Main effects plot for roughness parameter *S_a_*; (**b**) Interaction plot for roughness parameter *S_a_*.

More in detail, roughness *S_a_* is heavily affected from the depth of cut, the feed per tooth, and their interaction. Moreover, as observable from main effects and interaction plot in Figure 10, the higher the aforementioned process parameters or their interaction, the higher the roughness *S_a_*.

**Figure 11:**
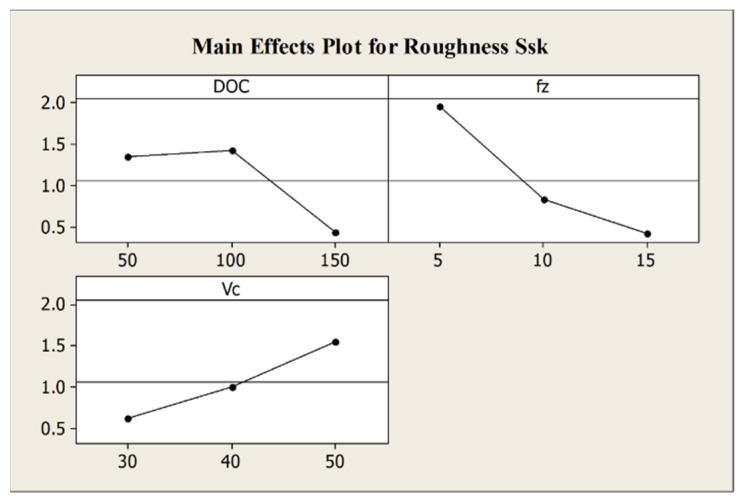
Main effects plot for roughness parameter *S_sk_*.

The feed per tooth strongly influences the roughness *S_sk_* while the depth of cut has only a marginal effect. Additionally, Figure 11 illustrates that the value of *S_sk_* decreases when the feed per tooth and the depth of cut increase, meaning that with a reduction of the process parameters a surface with more asperities is expected.

**Figure 12:**
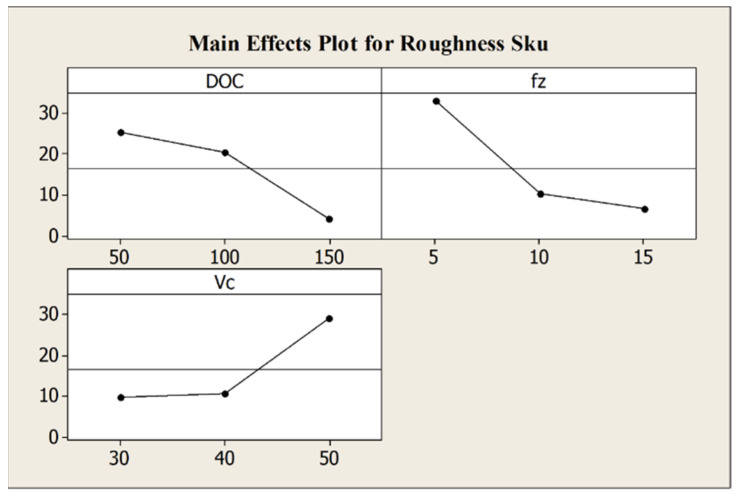
Main effects plot for roughness parameter *S_ku_*.

The influence of depth of cut and feed per tooth is confirmed from the analysis results for *S_ku_* as well, where the increase of the process parameters leads to a decrease of *S_ku_* value and, as a consequence, to a smoother surface. The main effects plot for *S_ku_* in Figure 12 shows this behavior.

The outcomes of the previously presented analyses indicates that the roughness *S_a_* must be taken into account to perform the optimization of the process parameters set. Hole and pin diameters, in fact, have good precision and their accuracy can be managed by adjusting their offsets respect to the required nominal values. Moreover, circularity and conicity of the pins do not compromise the mechanical coupling.

**Figure 13:**
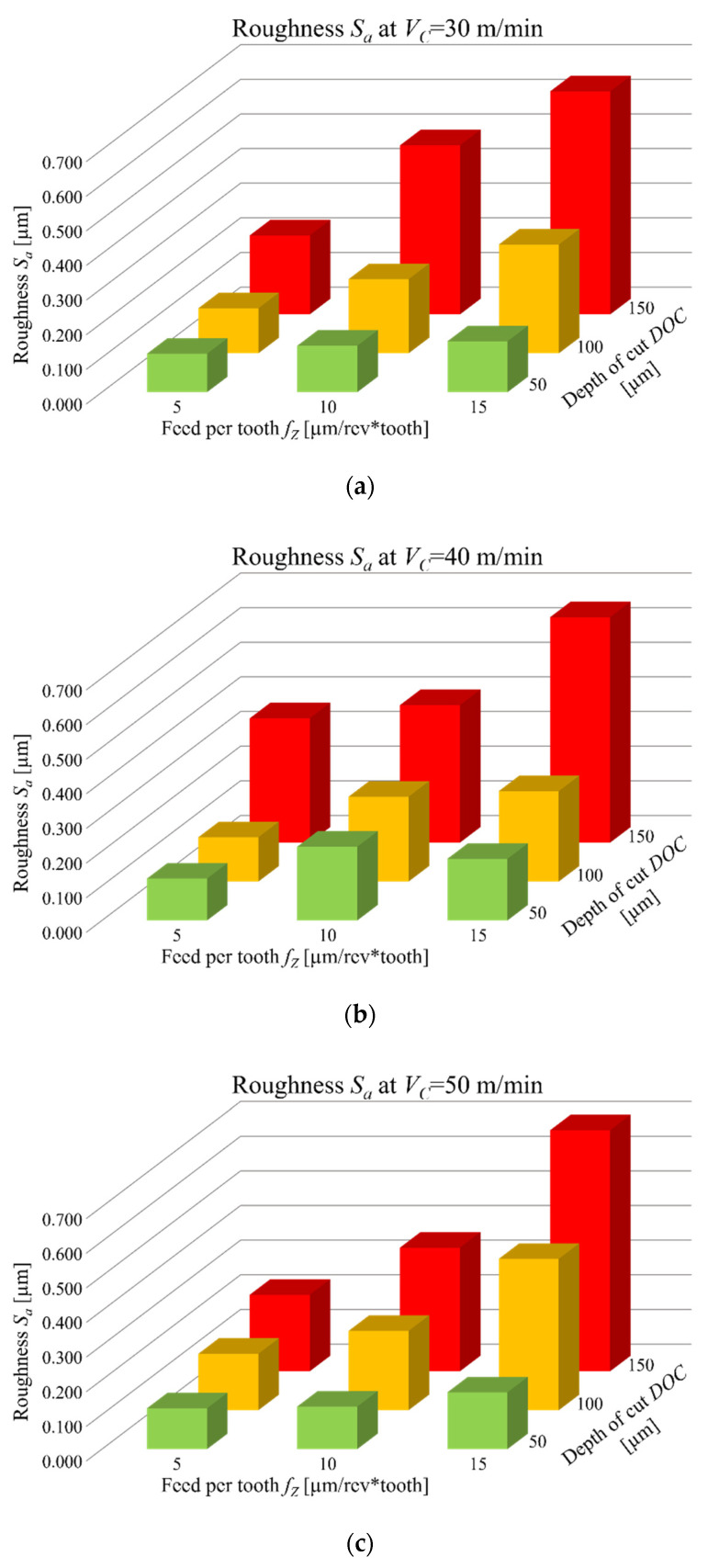
Roughness *S_a_* behavior as a function of *DOC* and *f_Z_*: (**a**) at 30 m/min; (**b**) at 40 m/min; (**c**) at 50 m/min.

The variation of roughness *S_a_* ranges from a minimum value of 0.111 µm to a maximum value of 0.696 µm. *S_a_* measurements are summarized in Figure 13, where they are distributed in three graphs corresponding to the three different cutting speeds.

**Figure 14:**
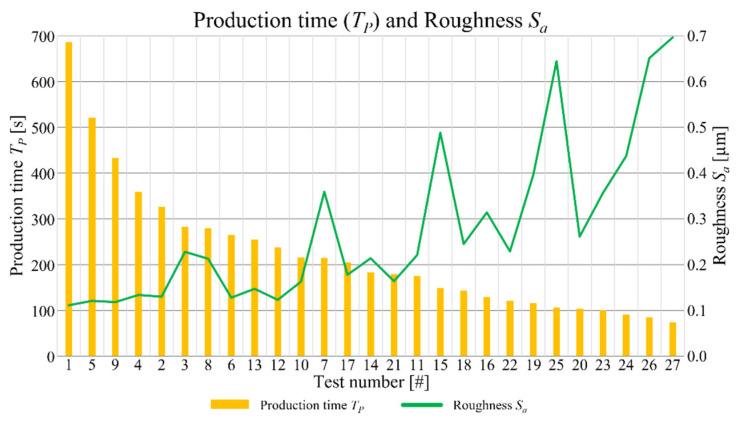
Production time (*T_P_*) and roughness *S_a_* as a function of the experimental tests.

As already underlined, low values of depth of cut and feed per tooth, that have the greatest influence on *S_a_*, lead to the best surface quality, thus the lowest process parameters values should be employed to achieve high levels of finishing. On the other hand, the process optimization must warrant an acceptable production time (*T_P_*). In Figure 14 the correlation between roughness *S_a_* and production time for the performed tests is showed.

For a better interpretation of the graph in Figure 14, the order of the experimental tests is rearranged from the one requesting the highest production time to the one requiring the lowest. The trend underlined by the graph is that reducing the production time a grow of the roughness is detected. Hence, a compromise between them is necessary, in order to optimize the process parameters selection. Tests 17, 14, and 21 individuate the best deal. Related to these tests, the roughness *S_a_* varies between 0.164 and 0.214 µm while the production time ranges amongst 179 s and 205 s. Tests 17 and 21 were both accomplished employing the minimum value of the depth of cut (*DOC* = 50 µm) and the maximum value of the feed per tooth (*f_Z_* = 15 µm) leading to a pin diameter that is far from the nominal value of 0.600 mm with, in addiction, an irregular shape. Moreover, the hole diameter for the Test 17 is outside the band of the measurement accuracy (Figure 4), meaning a loss of precision for this combination of parameters. Finally, considering the good compromise between production time and final surface roughness, and the compliance of dimensional and geometrical tolerances, the outcomes of this optimization reveals that the optimized process parameters are those of Test 14. This test is characterized by the application of central level values of both feed per tooth (*f_Z_* = 10 µm) and depth of cut (*DOC* = 100 µm) at the lowest cutting speed (*V_C_* = 30 m/min), providing a pin diameter of 600.8 + 2.5 µm, with low values of circularity (19.1 µm) and conicity (24%), a roughness *S_a_* equal to 0.214 µm and a production time equal to 183 s. A reduction of feed per tooth and depth of cut, maintaining the lowest cutting speed, shows a better value of surface roughness than the one of Test 14, but the production times are higher than the acceptable value of 283 s. On the other hand, keeping at low cutting speed value and using the highest ones for the other parameters, leads to quick production times, but to a compromised roughness. This can be explained considering the elevated chip cross-sectional area and the related high cutting energy required. The increase of this latter, generates higher cutting temperatures, favoring a ductile chip formation, rather than a brittle one, that decreases the surface quality.

In order to validate the entire procedure of optimization, a functioning wristwatch movement was fabricated in the laboratories of the University of Brescia. A first set of main plate and bridges were machined by using the process parameters suggested by a catalogue. The result is not considerable acceptable due to several issues:

- The workpieces profiles show large burrs that compromises the mechanical coupling between the bridges and the main plates and between the machined parts and the jewels;
- Pins show bulging distortion and the cylindricity tolerance is not respected;
- Micro-holes circularity is not adequate;
- The surface roughness is elevated, and it affects the movement functionality.

**Figure 15:**
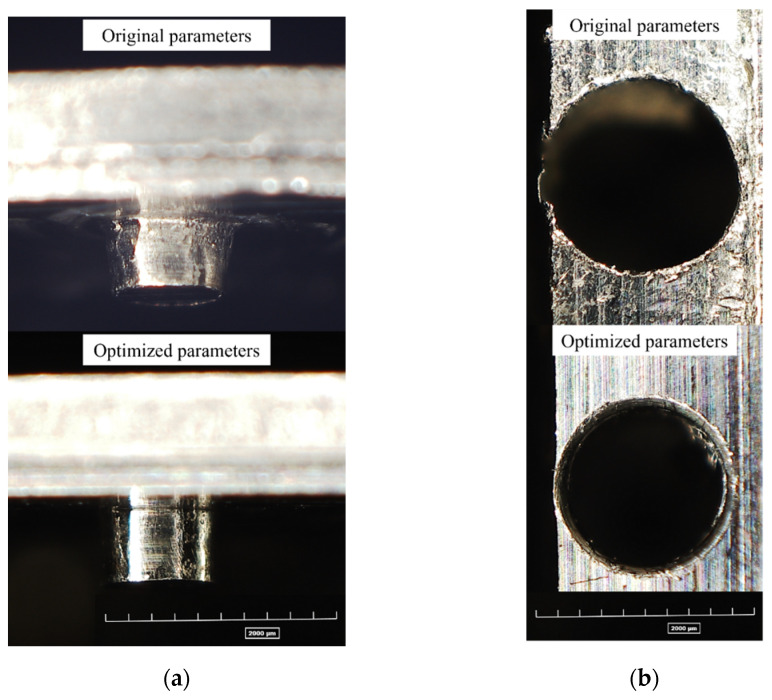
Results comparison between original and optimized process parameters for: (**a**) pin conicity; (**b**) hole diameter and circularity.

**Figure 16:**
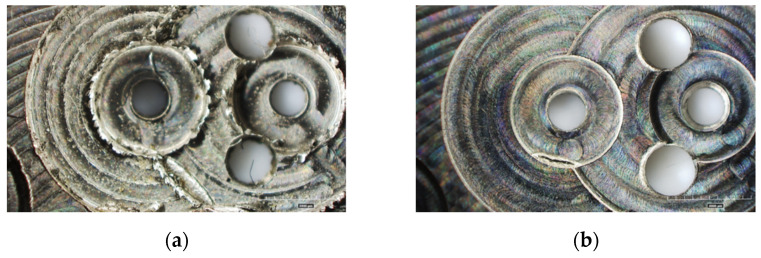
Comparison between the features machined with: (**a**) original process parameters; (**b**) optimized process parameters.

The same parts were subsequently manufactured by employing the optimized process parameters, id est a depth of cut *DOC* = 100 µm, a feed per tooth *f_Z_* = 10 µm, and a cutting speed *V_C_* = 30 m/min, were tested by the machining of a new main plate and bridges. The quality of results considerably increases as visible in Figure 15 and Figure 16. The new pins on the movement do not exhibit bulging effects and reveals good circularity (Figure 15a). This latter is excellent for the new radial holes as well (Figure 15b). The burrs observable in the original main plate are totally absent in the new configuration, and the regularity of the holes guarantees the correct jewels assembly (Figure 16).

The quality characteristics of the realized features resulted to be improved by the application of the optimized process parameters respect to the ones suggested by the tool catalogue, revealing the functionality of the proposed optimization method. For completeness, it is important to underline that the tool geometry employed in the experimental campaign is unique, and the variability window of the process parameters is ascribable to finishing operations. Hence, in the case of a different tool geometry, and operation, such as roughing, the employment of the identified optimized parameters should not be applicable. Due to the prominent nature of size and surface effects in micromachining, the modification of ratio between uncut chip thickness and tool edge radius alters the chip formation mechanism, varying CuNi18Zn20 machinability. In order to select the most suitable process parameters, when machining setup differs from the one employed in this work, this behavior must be considered.

## 4. Conclusions

The paper described an experimental approach to a problem of optimization of cutting process parameters in a complex micro-machining case. As results of the experimental campaign of 27 prototypes machining, it was evidenced that the employment of the highest depth of cut and feed per tooth induced to excessive burrs distribution. Moreover, the final geometry showed irregularities in the tests with *f_Z_* = 10 µm combined with *V_C_* = 40–50 m/min, and *f_Z_* = 15 µm unrelatedly the cutting speed. A critical size for the machining of microfeatures was individuated as 1 mm. The features in submillimeter dimensional scale requires machining with low feed rates and low cutting speeds.

The conicity of pins does not result a critical tolerance while a lack of accuracy emerged about the offset between the nominal and the effective diameters of pins and holes. The size of the deviation does not depend on the process parameters and it is equal to the 1.5% of the nominal diameter. A possible cause is the micro mill run-out due to the evidence that the holes show higher then expected diameters. Vice versa, the pins have lower then expected diameters due to tool eccentricity. A strong correlation between roughness and depth of cut emerged by the data analysis. The trend is not commonly reported in conventional scale machining. The chatter vibrations were identified as a possible explanation of this scale effect. The depth of cut increasement implicates higher cutting forces which can determines the vibrations of the workpiece. The roughness resulted directly correlated also to the feed rate.

## Figures and Tables

**Table 1:** Features of micromill SECO 905L008-MEGA-T.

Propriety	Value
Manufacturer	SECO
Code	905L008-MEGA-T
Nominal diameter [μm]	800
Number of flutes	2
Measured diameter [μm]	795 ± 1
Measured cutting-edge radius [μm]	6 ± 0.8
Helix angle [°]	20
Rake angle [°]	4
Material	Tungsten Carbide
Material coating	Titanium Nitride

**Table 2:** List of the values of the micromilling process parameters.

Process Parameter	Value A	Value B	Value C
Depth of cut *DOC* [μm]	50	100	150
Feed per tooth *f_Z_* [μm/rev*tooth]	5	10	15
Cutting speed *V_C_* [m/min]	30	40	50

**Table 3:** List of the process parameters and production time.

Test	*DOC* [μm]	*f_Z_* [μm/rev*t]	*V_C_* [m/min]	Time [s]	Test	*DOC* [μm]	*f_Z_* [μm/rev*t]	*V_C_* [m/min]	Time [s]
1	50	5	30	686	15	150	10	30	149
2	100	5	30	326	16	100	15	30	129
3	150	5	30	283	17	50	15	40	205
4	50	10	30	359	18	100	10	40	143
5	50	5	40	521	19	150	10	40	116
6	100	5	40	265	20	100	15	40	104
7	150	5	40	215	21	50	15	50	179
8	50	10	40	280	22	100	10	50	121
9	50	5	50	433	23	150	10	50	100
10	100	5	50	216	24	100	15	50	91
11	150	5	50	175	25	150	15	30	106
12	50	10	50	238	26	150	15	40	85
13	50	15	30	255	27	150	15	50	74
14	100	10	30	183					

**Table 4:** Analysis of variance of hole diameter *φ_H_* = 1.000 mm.

Source	DoF	Seq SS	Adj SS	Adj MS	F	P
*DOC*	2	0.0002108	0.0002108	0.0001054	5.20	0.036
*f_z_*	2	0.0001415	0.0001415	0.0000707	3.49	0.081
*V_C_*	2	0.0000077	0.0000077	0.0000039	0.19	0.830
DOC*f_z_	4	0.0003732	0.0003732	0.0000933	4.60	0.032
DOC*V_C_	4	0.0001228	0.0001228	0.0000307	1.51	0.286
f_z_*V_C_	4	0.0000782	0.0000782	0.0000196	0.96	0.477
Error	8	0.0001622	0.0001622	0.0000203		
Total	26	0.0010965				

**Table 5:** Analysis of variance of pin diameter *φ_P_* = 0.600 mm.

Source	DoF	Seq SS	Adj SS	Adj MS	F	P
*DOC*	2	0.0001344	0.0001344	0.0000672	1.56	0.269
*f_z_*	2	0.0001940	0.0001940	0.0000970	2.24	0.168
*V_C_*	2	0.0000248	0.0000248	0.0000124	0.29	0.758
DOC*f_z_	4	0.0001173	0.0001173	0.0000293	0.68	0.626
DOC*V_C_	4	0.0000915	0.0000915	0.0000229	0.53	0.718
f_z_*V_C_	4	0.0000344	0.0000344	0.0000086	0.20	0.932
Error	8	0.0003457	0.0003457	0.0000432		
Total	26	0.0009421				

**Table 6:** Analysis of variance of pin circularity.

Source	DoF	Seq SS	Adj SS	Adj MS	F	P
*DOC*	2	0.0001792	0.0001792	0.0000896	2.84	0.117
*f_z_*	2	0.0003530	0.0003530	0.0001765	5.59	0.030
*V_C_*	2	0.0001263	0.0001263	0.0000631	2.00	0.198
DOC*f_z_	4	0.0005390	0.0005390	0.0001347	4.26	0.039
DOC*V_C_	4	0.0001299	0.0001299	0.0000325	1.03	0.449
f_z_*V_C_	4	0.0000988	0.0000988	0.0000247	0.78	0.568
Error	8	0.0002528	0.0002528	0.0000316		
Total	26	0.0016789				

**Table 7:** Analysis of variance of pin conicity.

Source	DoF	Seq SS	Adj SS	Adj MS	F	P
*DOC*	2	0.00415	0.00415	0.00208	0.16	0.852
*f_z_*	2	0.02214	0.02214	0.01107	0.87	0.455
*V_C_*	2	0.01105	0.01105	0.00553	0.43	0.662
DOC*f_z_	4	0.01823	0.01823	0.00456	0.36	0.831
DOC*V_C_	4	0.05746	0.05746	0.01437	1.13	0.407
f_z_*V_C_	4	0.03742	0.03742	0.00936	0.74	0.593
Error	8	0.10167	0.10167	0.01271		
Total	26	0.25214				

**Table 8:** Analysis of variance of roughness parameter *S_a_*.

Source	DoF	Seq SS	Adj SS	Adj MS	F	P
*DOC*	2	0.437391	0.437391	0.218696	104.56	0.000
*f_z_*	2	0.204669	0.204669	0.102335	48.93	0.000
*V_C_*	2	0.001188	0.001188	0.000594	0.28	0.760
DOC*f_z_	4	0.096272	0.096272	0.024068	11.51	0.002
DOC*V_C_	4	0.012273	0.012273	0.003068	1.47	0.298
f_z_*V_C_	4	0.015411	0.015411	0.003853	1.84	0.214
Error	8	0.016732	0.016732	0.002092		
Total	26	0.783938				

**Table 9:** Analysis of variance of roughness parameter *S_sk_*.

Source	DoF	Seq SS	Adj SS	Adj MS	F	P
*DOC*	2	5.64	5.64	2.82	1.60	0.261
*f_z_*	2	11.714	11.714	5.857	3.32	0.049
*V_C_*	2	3.945	3.945	1.972	1.12	0.374
DOC*f_z_	4	2.646	2.646	0.662	0.37	0.821
DOC*V_C_	4	5.827	5.827	1.457	0.82	0.545
f_z_*V_C_	4	2.894	2.894	0.723	0.41	0.797
Error	8	14.132	14.132	1.766		
Total	26	46.797				

**Table 10:** Analysis of variance of roughness parameter *S_ku_*.

Source	DoF	Seq SS	Adj SS	Adj MS	F	P
*DOC*	2	917.7	1249	624.5	4.32	0.033
*f_z_*	2	890.2	976.9	488.4	3.38	0.062
*V_C_*	2	2157.5	2157.5	1078.8	1.39	0.303
DOC*f_z_	4	1566.9	1566.9	391.7	0.51	0.733
DOC*V_C_	4	2382.6	2382.6	595.7	0.77	0.574
f_z_*V_C_	4	2393.8	2393.8	598.5	0.77	0.572
Error	8	6195.3	6195.3	774.4		
Total	26	20,667.8				
